# Trends over time in enrollment in non-group health insurance plans by tobacco use in the United States

**DOI:** 10.1016/j.pmedr.2017.05.010

**Published:** 2017-05-17

**Authors:** Michael F. Pesko, Johanna Catherine Maclean, Cameron M. Kaplan, Steven C. Hill

**Affiliations:** a Weill Cornell Medicine, Department of Healthcare Policy and Research, New York, NY, United States; b Temple University, Department of Economics, Philadelphia, PA, United States; c University of Tennessee Health Science Center, Department of Preventive Medicine, Memphis, TN, United States; d Agency for Healthcare Research and Quality, Center for Financing, Access and Cost Trends, Rockville, MD, United States

**Keywords:** Healthcare.gov, Health insurance, Individual marketplace, Nongroup health insurance, Tobacco use, Tobacco products

## Abstract

Healthcare.gov was created to facilitate the market for non-group insurance in states that did not establish their own marketplaces. In Healthcare.gov, families are asked to report their tobacco use status, and tobacco use surcharges of up to 50% may result. We tabulate enrollment information for 35 states offering insurance plans through Healthcare.gov in both 2014 and 2016. The Centers for Medicare and Medicaid Services provided counts of enrollees indicating tobacco use, by state, year, and risk level. The number of enrollees increased from 5.0 million in 2014 to 9.4 million in 2016. From 2014 to 2016, the number of enrollees rose 39% for tobacco users and 90% for non-tobacco users. Reported non-tobacco user enrollment rose faster than reported tobacco user enrollment in 30 out of 35 states. Reported tobacco users are enrolling in marketplace plans at a lower rate and are more likely to enroll in less generous plans. The decline in smoking as reported when purchasing insurance on Healthcare.gov surpasses declines in smoking observed in other data sources, which suggests that tobacco users may be decreasingly likely to report their tobacco use status accurately to avoid surcharges. Finally, we find no evidence of the surcharges being associated with lower enrollment among self-reported tobacco users, or in rates of smoking.

## 1 Introduction

Tobacco use is associated with over 400,000 annual U.S. deaths (US Department of Health and Human Services, 2014) and one third of all U.S. cancer deaths (Danaei et al., 2009). Health insurance may help tobacco users access effective smoking cessation treatments and, in turn, quit tobacco use. The Affordable Care Act (ACA) improved insurance access overall by establishing marketplaces, which simplify purchasing non-group insurance, and offering premium tax credits that can reduce insurance costs for lower income families. Healthcare.gov was created to facilitate purchasing non-group health insurance for individuals or families.

The ACA limits the factors insurance issuers in the individual market could use in setting premiums to geographic areas, age, family size, and tobacco use. Previously, insurers could use health status to set premiums; insurers could charge sicker people more than healthy people. Health rating was banned by the ACA, but tobacco rating of up to 50% remained a criterion that could be used to differentiate premiums. While charging tobacco users more for health insurance may reduce the moral hazards of tobacco use and provide a financial incentive to quit, tobacco rating may also cause health insurance to be unaffordable to smokers and reduce their access to healthcare services and products that could help them to quit successfully.

Consumers enrolling on Healthcare.gov provide information about their tobacco use and also select a health insurance plan. Consumers are instructed to report whether each adult applying for coverage has used tobacco on average four or more times per week over the past six months, which classifies them as a tobacco user. Insurance plans' actuarial values are represented by different metal levels including platinum, gold, silver, bronze, and catastrophic, with platinum plans having the least cost sharing (but highest premiums) and catastrophic plans providing the most cost sharing (but lowest premiums). Catastrophic plans are only available to people under 30 or meeting a hardship exemption. Consumers who qualify for cost-sharing reductions based on their income must select a silver plan to receive these subsidies (US Centers for Medicare & Medicaid Services).

On average, tobacco use surcharges are roughly 15% above the premiums of non-tobacco users (Kaplan et al., 2014, Liber et al., 2015). Premium tax credits do not apply to tobacco use surcharges, making marketplace enrollment prohibitively expensive for some tobacco users (Hill, 2015). One study suggests high tobacco use surcharges reduced marketplace enrollment in 2014 by 11.6 percentage points compared to no surcharges (Friedman et al., 2016). However, the maximum penalty for not reporting tobacco use is a bill for the unpaid surcharges; therefore, there appears to be little financial incentive for smokers to accurately report their tobacco use.

Using aggregate enrollment data from Healthcare.gov, we study how enrollment has changed for individuals reporting the use of tobacco versus non-users. We further explore the influence of the tobacco use surcharge levels on growth in marketplace enrollment by tobacco use status, as well as changes in smoking rates.

## 2 Methods

Aggregate Healthcare.gov enrollment data for the federally facilitated marketplace (FFM) and state-partnership marketplace (SPM) for 2014 and 2016 was obtained through a Freedom of Information Act (FOIA) request. Specifically, the Centers for Medicare and Medicaid Services (CMS) provided counts of the numbers of enrollees indicating tobacco use, by state, year, and risk level. We calculated the number of non-tobacco users by subtracting the number of tobacco users from total enrollees. For 2014, CMS also provided total enrollees by state and metal level. For year 2016, we obtained total enrollees for each state and the percent of total enrollees in each state's metal level from a publication (US Department of Health and Human Services, 2016). Totals reflect enrollment at the end of the open enrollment period. Additional details on how we aggregated the data are provided in online supplementary materials.

We did not receive data from our FOIA request for states participating in state-based marketplaces (SBM). For the SBM states, we lacked data in both years (CA, CO, CT, DC, KY, MD, MA, MN, NY, RI, VT, WA), in only 2014 (HI, NV, OR), or in only 2016 (ID). We used data for the other 35 states participating in the FFM and SPM in both years, which provided a consistent sample of states throughout the study period.

We do not perform statistical testing of our enrollment data because we have censuses.

We used two survey data sources to compare rates of smoking in Healthcare.gov with rates of smoking among the individuals most likely to use Healthcare.gov. We used both the Behavioral Risk Factor Surveillance System (BRFSS) and National Health Interview Survey (NHIS) for years 2014 to 2015 to compare changes in reported tobacco use in these surveys with that reported on Healthcare.gov (2016 data is not yet available for these surveys).

The BRFSS is a nationally representative telephone-based survey that collects information on health-related risk behaviors, chronic health conditions, and use of preventive services. The large sample size (> 400,000 in each year) of BRFSS supports state estimates, and state identifiers are publicly available, allowing us to estimate smoking prevalence in the 35 states in our study. The sample that we use is nonelderly respondents age 25 to 64 in the 35 states who reported their smoking status and reported annual incomes above $20,000, since these are the people most likely to be in the market for non-group health insurance through Healthcare.gov.

The NHIS conducts in-person household interviews to provide data to track health status, health care access, and progress toward achieving national health objectives. While we do not have access to state-specific information in NHIS, the information on health insurance is more detailed than that provided by BRFSS, and we use it to identify rates of smoking nationally among individuals between the ages of 18–64 purchasing private insurance through exchanges. For both data sources, we use population weights to calculate smoking rates.

## 3 Results

**Fig. 1:**
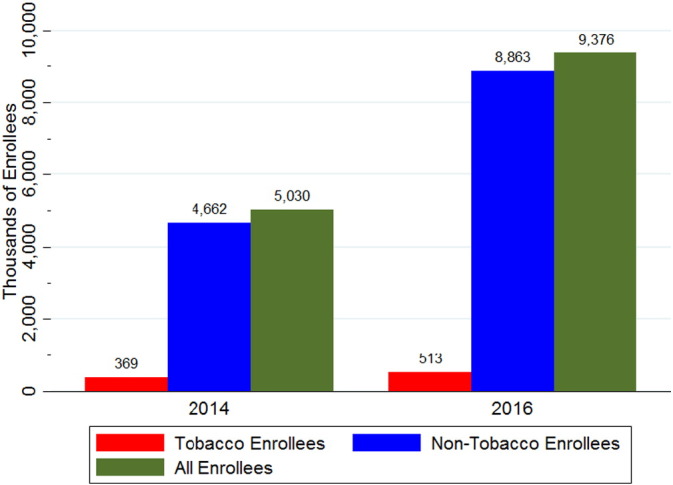
Marketplace enrollment by tobacco users and non-tobacco users in 2014 and 2016.

Source: Authors' analysis of Healthcare.gov 2014 and 2016 enrollment data.

Enrollment on Healthcare.gov across these 35 states increased from 5.03 million in 2014 to 9.38 million in 2016, an 86% increase (Fig. 1). In 2014, 369,000 enrollees were tobacco users, rising to 513,000 in 2016, an increase of 39%. In contrast, enrollment for non-tobacco users rose 90% over this time period. As a result, tobacco-using enrollees fell from 7.3% to 5.5% of all enrollees between 2014 and 2016.

Table 1 shows the percent change in enrollment from 2014 to 2016 by state and tobacco use. Two states (Arizona and Pennsylvania) had fewer tobacco users in 2016 than in 2014, and 3 states (Iowa, Nebraska, and Wyoming) had more than twice as many tobacco users. In contrast, 14 states more than doubled non-user enrollment. Table 1 also shows the minimum effective surcharge for 2014 for tobacco users purchasing bronze plans. The tobacco use surcharges ranged from 0% to 46% of total premiums. Finally, Table 1 shows the smoking rate decline (3.5% decline, *p* < 0.05) in the 35 states for the population of nonelderly respondents age 25 to 64 who reported their smoking status and reported annual incomes above $20,000. This 3.5% decline in smoking, or 0.7 percentage point decline, was smaller than the 2.2 percentage point decline in reported tobacco users as a share of Healthcare.gov enrollment. As a sensitivity analysis, we also estimated the change in smoking rate from 2014 to 2015 in the same sample, but further restricted to those with health insurance. The year-to-year decline among the insured was similar to that of the larger sample (both with and without health insurance) and was not statistically significant.

**Table 1:** Growth in individual marketplace enrollment, surcharge levels, and changes in smoking from 2014 to 2016.

State	(1)	(2)	(3)	(4)	(5)
	Growth in tobacco user enrollment, 2014–16	Growth in non-tobacco user enrollment, 2014–16	Ratio: (1) / (2)	Mean minimum effective surcharge for bronze plans, 2014	Change in smoking status, 2014–15^1^
AK	93.98%	81.51%	1.15	0%	− 3.85%
AL	50.24%	100.85%	0.50	10.00%	7.24%
AR	24.67%	67.32%	0.37	0.80%	− 5.70%
AZ	− 11.96%	64.96%	− 0.18	0.91%	− 8.14%
DE	75.54%	100.21%	0.75	10.00%	− 3.57%
FL	43.75%	98.62%	0.44	15.48%	− 10.77%
GA	49.90%	110.24%	0.45	14.83%	12.24%
IA	100.64%	103.79%	0.97	21.87%	− 3.89%
IL	35.28%	77.20%	0.46	21.03%	− 9.79%
IN	16.33%	56.32%	0.29	29.74%	− 15.31%⁎
KS	50.58%	88.28%	0.57	19.93%	7.34%
LA	66.41%	146.63%	0.45	19.90%	− 6.33%
ME	72.06%	84.26%	0.86	1.44%	− 1.19%
MI	3.66%	47.14%	0.08	8.48%	5.40%
MO	65.04%	109.07%	0.60	21.14%	11.57%
MS	25.64%	93.17%	0.28	13.15%	5.29%
MT	76.50%	60.34%	1.27	15.00%	− 1.68%
NC	51.54%	92.28%	0.56	20.00%	− 6.78%
ND	46.94%	71.22%	0.66	14.26%	− 4.17%
NE	127.49%	115.92%	1.10	37.93%	− 2.02%
NH	32.26%	49.01%	0.66	30.22%	− 6.07%
NJ	21.06%	63.96%	0.33	0%	− 11.49%
NM	40.79%	77.85%	0.52	11.09%	− 7.41%
OH	39.94%	63.90%	0.63	19.14%	8.54%
OK	49.81%	121.58%	0.41	21.69%	12.30%
PA	− 0.04%	39.86%	0.00	14.88%	− 11.12%
SC	7.81%	143.07%	0.05	4.17%	− 12.73%⁎
SD	92.03%	100.25%	0.92	19.89%	5.70%
TN	54.63%	116.19%	0.47	15.00%	− 6.05%
TX	48.30%	108.02%	0.45	20.79%	− 2.13%
UT	69.40%	107.18%	0.65	12.97%	− 6.19%
VA	68.66%	106.87%	0.64	17.25%	− 11.91%⁎
WI	34.59%	72.55%	0.48	16.86%	− 0.43%
WV	85.85%	96.34%	0.89	10.20%	4.51%
WY	101.47%	92.17%	1.10	46.37%	7.19%
ALL	39.02%	90.11%	0.43	15.92%	− 3.50%⁎

For each county and age, the minimum effective surcharge is how much more expensive the lowest cost plan is for a tobacco user relative to a nonuser. The average is weighted by county population, with each age 21 to 64 equally weighted (US Department of Health and Human Services, 2014).

1 Among a sample of nonelderly respondents age 25 to 64 who reported their smoking status and reported annual incomes above $20,000.

⁎ Significant at the 5% level.

We hypothesize that the minimum effective surcharge may have reduced smoking and reduced enrollment in marketplace plans by self-reported tobacco users. To explore this hypothesis, we correlate (with population adjustment) the tobacco use surcharge level (Table 1, column 4) with the growth in tobacco use enrollment (column 1) or the ratio of growth in tobacco user enrollment to non-user enrollment (column 3). Neither correlation provided evidence to support our hypothesis that higher surcharges reduced reported tobacco user enrollment. Additionally, the surcharge was not correlated with a decline in the rate of smoking (column 5), which may suggest that the surcharges are not an effective mechanism to reduce tobacco use.

In unreported results from the NHIS, we found that the smoking rate among individuals between the ages of 18–64 purchasing private health insurance through exchanges was 13.2% in 2014 and 14.8% in 2015. While these numbers are national estimates and are not specific to the 35 states using Healthcare.gov in both years, they do suggest significant underreporting of smoking on Healthcare.gov and no apparent decline in smoking rates in the early years (in contrast to a significant decline on Healthcare.gov). Unfortunately, the public NHIS data does not contain state identifiers that would permit us to explore how the 2014 surcharge levels correlated with changes in smoking in this market.

In Online Fig. 1, we show the percent in each metal level by year for tobacco users and non-users. In both years, tobacco users were more likely to enroll in catastrophic and bronze plans than non-tobacco users, and were less likely to enroll in gold, platinum, and silver plans than non-tobacco users. For both tobacco users and non-users, there was a sharp decline in the share of enrollment in gold and platinum plans in 2016 compared to 2014. The share of enrollment in silver plans remained stable for non-users and declined by 1.5 percentage points for users. The share of enrollment in bronze and catastrophic plans increased for both groups. Thus, while both users and non-users shifted to less generous metal levels, tobacco users remained more likely to be in less generous metal levels in 2016.

## 4 Discussion

While total marketplace enrollment has increased since 2014, reported tobacco users' enrollment has not kept pace with overall enrollment. Consequently, tobacco users as a percent of all enrollees have declined over time. Prior to the ACA, tobacco users were more likely to be uninsured and underinsured (Wilper et al., 2009). To the extent that our data reflect that actual tobacco users are enrolling in Healthcare.gov at a lower growth rate than non-tobacco users, and in less generous plans, then disparities for tobacco users in health insurance coverage and in the generosity of that coverage are widening. Those tobacco users who did enroll were more likely to choose plans with lower metal levels. Lower-income tobacco users who selected bronze instead of silver plans missed the cost sharing reductions available to silver plan enrollees (and higher) with incomes from 100% to 250% of poverty.

However, the finding of smoking rates being stable in exchange plans from 2014 to 2015 in NHIS data suggests that perhaps tobacco users are underreporting their tobacco use on Healthcare.gov, rather than not enrolling. If smokers are concealing their smoking behaviors to avoid the surcharges, this could create the perverse incentive to not discuss their tobacco use with healthcare professionals, since doing so may result in the health insurance company being billed for tobacco cessation counseling and the health insurance company retroactively applying the tobacco use surcharge. If tobacco use surcharges remain part of the individual market going forward, we argue that validation methods beyond self-report are needed in order to not create perverse incentives that may discourage smokers from receiving medical care to help them quit using tobacco.

One study suggests high tobacco use surcharges reduced marketplace enrollment in 2014 by 11.6 percentage points compared to no surcharges (Friedman et al., 2016); however, in our study we do not find a relationship between the surcharges and self-reported smoking. One possible difference between these results is that we use only variation in the surcharge level in year 2014, since surcharges were used prior to 2014 in most states (The Henry J. Kaiser Family Foundation, 2012) and to the best of our knowledge no systematic, state-level data exists on the size of these surcharges prior to 2014. While a limitation of our using Healthcare.gov data is that higher surcharges may not only decrease actual tobacco use but increase concealing of tobacco use, we do not find evidence of either occurring disproportionately in places with higher surcharges.

Providing equitable access to affordable health care for tobacco users and non-users, two groups with different risks for high healthcare costs, is a challenge for policymakers. On one hand, tobacco surcharges burden smokers by increasing premiums (Friedman et al., 2016). We found surcharges are also associated with selecting less generous health insurance plans. On the other hand, smokers tend to have more expensive health care use (Kaplan et al., 2014, Moriarty et al., 2012, Sturm, 2002, Cowan and Schwab, 2011). Actuarial studies indicate that if surcharges were limited or eliminated, then non-tobacco users would be charged higher premiums to cover the additional costs of tobacco users (Dillon, 2013).

Our findings remain relevant to current, ongoing debates about the nongroup health insurance market in the United States. The ACA did not create tobacco rating; in fact, prior to the ACA, tobacco rating was allowed in all but six states in the individual marketplace (The Henry J. Kaiser Family Foundation, 2012). Therefore, tobacco rating would remain relevant in a possible post-ACA world.

## 5 Conclusion

Little was previously known about tobacco users' experiences in Healthcare.gov as plans have changed over the first three years. We found that, from 2014 to 2016, the number of enrollees in individual marketplace plans rose 39% for reported tobacco users and 90% for reported non-tobacco users. Additionally, reported tobacco users were more likely than non-tobacco users to select bronze plans, which have less generous benefits than silver, gold and platinum plans. The tobacco use surcharge level was not correlated with slower growth of tobacco user enrollment or decreases in smoking. The rapid decline of tobacco user enrollment from 2014 to 2016, compared to relatively stable rates of smoking for individuals enrolling in exchange-based health insurance in 2014 and 2015, as reported by NHIS, suggests that tobacco users may be concealing their smoking behaviors on Healthcare.gov. Future work is needed to document the extent of concealment and to assess the potential effects of concealing behavior on access to medical resources to quit tobacco use.

## Funding statement

This work was supported by the American Cancer Society grant number RSGI-16-019-01.

## Conflicts of interest statement

Dr. Hill is employed by the Agency for Healthcare Research and Quality. The views expressed in this paper are the authors' and do not reflect the views of the Agency for Healthcare Research and Quality or the U.S. Department of Health and Human Services. There are no other conflicts of interest to report.

## Contributorship statement

Dr. Pesko submitted the Freedom of Information Act request and obtained the data. All authors were involved with the data analysis, interpretation of the results, and writing/editing of the paper.

## Appendix A Supplementary material

Supplementary material Image 1
