# Supplementary material for: Trends over time in enrollment in non-group health insurance plans by tobacco use in the United States
Source: Prev Med Rep. 2017 May 17;7:46–9. doi: 10.1016/j.pmedr.2017.05.010 (PMC5447394; doi:10.1016/j.pmedr.2017.05.010)
Supplement: Supplementary file 1 — Supplementary material [file mmc1.pdf]

Online Materials

Methods Appendix

We were unable to locate data on total enrollees by state-metal level for 2015 that was measured comparably to our tobacco enrollment data. Effectuated enrollment for 2015 was available from an outside source,[1] but our data from CMS were for total enrollees at the end of the open enrollment period, not total effectuated enrollees, which excludes those who did not pay their premiums. Additionally, county-metal level enrollment data were available from another outside source,[2] but high rates of missing data (due to CMS efforts to mask small cells and therefore provide privacy protection to consumers) in this outside data reduced our ability to use these outside data with our FOIA data. Because we could not locate comparable and complete data on total enrollment by state and metal level for 2015, we use years 2014 and 2016 only in our analysis.

We used a variety of imputation strategies to address missing or imprecise enrollment data. CMS did not release enrollment cell counts below 12. This resulted in missing total enrollees in 2 state-metal combinations in 2014, missing tobacco enrollees in 18 state-metal combinations in 2014, and missing tobacco enrollees in 2 state-metal combinations in 2016. We imputed the value 12 for the 2 states with missing total enrollees for year 2014. Out of >5 million overall enrollees in year 2014, our choice of imputing for these 2 states (24 enrollees) appears insignificant. For tobacco users we calculated upper bound estimates that imputed missings with a value of 12 and lower bound estimates that imputed missings with a value of 0. Our results were stable across imputation methods, thus we show results using zero tobacco enrollees in these state-metal combinations.

Unlike 2014, we did not receive data from CMS on total enrollees for 2016. For 2016, we obtained total enrollees for each state and the percent of total enrollees in each state's metal level

---

[1] Center for Medicare & Medicaid Services. March 31, 2015 Effectuated Enrollment Snapshot [cited 2016 September 8. Available from: https://www.cms.gov/Newsroom/MediaReleaseDatabase/Fact-sheets/2015-Fact-sheets-items/2015-06-02.html.

[2] Center for Medicare & Medicaid Services. Qualifying Health Plan Selections by Metal Level and County, as of February 22, 2015 2015 [cited 2016 September 8]. Available from: https://data.cms.gov/Marketplace-Qualified-Health-Plan-QHP-/2015-Qualifying-Health-Plan-Selections-by-Metal-Le/x46j-d9yv.

from a published estimate.[3] In this source, these percentages were rounded to the nearest percentage point. The most common integer was 0%, which implies that fewer than half of a percent of the state's enrollees selected that metal level. In our main results, we imputed values for these plans as 0.25%; however, our results were not appreciably different if we instead used a lower bound of 0% or an upper bound of 0.49%.

[3] Department of Health and Human Services. Addendum to the Health Insurance Marketplaces 2016 Open Enrollment Period: Final Report Enrollment 2016 [cited 2016 Septebmer 8]. Available from: https://aspe.hhs.gov/sites/default/files/pdf/188026/MarketPlaceAddendumFinal2016.pdf.

Online Figure 1: Marketplace enrollment by metal level, year, and tobacco use status from 2014 and 2016

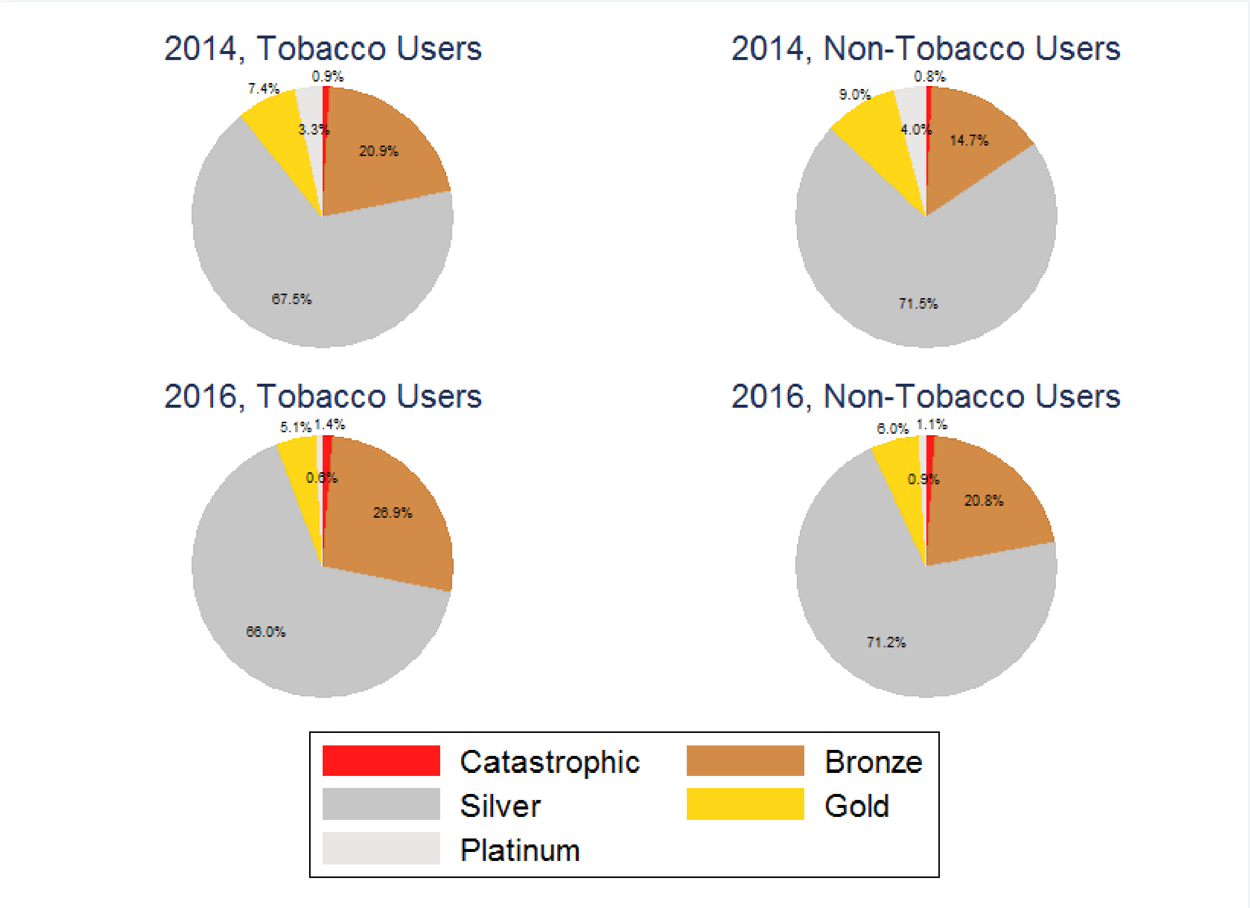

Source: Authors' analysis of Healthcare.gov 2014 and 2016 enrollment data.
